# The manifestation of affective symptoms in multiple sclerosis and discussion of the currently available diagnostic assessment tools

**DOI:** 10.1007/s00415-022-11359-6

**Published:** 2022-09-21

**Authors:** Melanie Filser, Axel Buchner, Gereon Rudolf Fink, Stefan M. Gold, Iris-Katharina Penner

**Affiliations:** 1 Department of Experimental Psychology, Heinrich Heine University, Düsseldorf, Germany; 2 Department of Neurology, University of Cologne, Cologne, Germany; 3 Institute of Neuroscience and Medicine (INM-3), Research Centre, Cognitive Neuroscience, Jülich, Germany; 4 Department of Psychiatry and Psychotherapy, Campus Benjamin Franklin (CBF), Charité Universitätsmedizin Berlin, Berlin, Germany; 5 Medical Department, Section Psychosomatics, Charité Universitätsmedizin Berlin, Berlin, Germany; 6 Institute of Neuroimmunology and Multiple Sclerosis (INIMS), Center for Molecular Neurobiology, University Medical Center, Hamburg-Eppendorf, Germany; 7 Department of Neurology, Medical Faculty, Heinrich Heine University, Düsseldorf, Germany; 8 COGITO Centre for Applied Neurocognition and Neuropsychological Research, Life Science Centre, Düsseldorf, Germany; 9 Department of Neurology, Inselspital, Bern University Hospital, University of Bern, Bern, Switzerland

**Keywords:** Multiple sclerosis, Affective symptoms, Mental health, Diagnostic assessment tools, Quality of life

## Abstract

**Introduction:**

In addition to physical and cognitive symptoms, patients with multiple sclerosis (MS) have an increased risk of experiencing mental health problems.

**Methods:**

This narrative review provides an overview of the appearance and epidemiology of affective symptoms in MS such as depression, anxiety, bipolar disorder, euphoria, and pseudobulbar affect. Furthermore, the association between affective symptoms and quality of life and the currently used diagnostic instruments for assessing these symptoms are considered whereby relevant studies published between 2009 and 2021 were included in the review.

**Results:**

Patients with mild and moderate disability more frequently reported severe problems with depression and anxiety than severe mobility problems. Apart from the occurrence of depression, little is known about the association of other affective symptoms such as anxiety, bipolar disorder, euphoria, and pseudobulbar affect and subsyndromal symptoms, which fail to meet the diagnostic criteria but are nevertheless a significant source of distress. Although there are a few recommendations in the research to perform routine screenings for diagnosable affective disorders, a standardized diagnostic procedure to assess subsyndromal symptoms is still lacking. As the applied measurements are diverse and show low accuracy to detect these symptoms, patients who experience affective symptoms are less likely to be identified.

**Discussion:**

In addition to the consideration of definite psychiatric diagnoses, there is an unmet need for a common definition and assessment of disease-related affective symptoms in MS. Future studies should focus on the improvement and standardization of a common diagnostic procedure for subsyndromal affective symptoms in MS to enable integrated and optimal care for patients.

## Background

Apart from physical symptoms, most patients with multiple sclerosis (MS) experience cognitive deficits [1] and affective symptoms which may negatively affect their social and family life, thereby contributing to the burden of the disease [2, 3]. Significantly, patients are found to experience severe affective symptoms at all levels of disease severity and patients with mild and moderate disability (EDSS 0–6.5) reported severe problems with depression and anxiety even more often than severe mobility problems [3].

The lifetime prevalence of affective disorders such as major depression or anxiety disorders in MS can be as high as 50% [4–6] and is thus substantially higher than in the general population [7, 8]. Several factors may mediate the occurrence of affective symptoms, including socioeconomic status, age, sex, and the region of residence. Even when these factors are controlled for, there remains a significantly higher annual prevalence risk of affective symptoms in patients with MS when compared to the general population [9]. While the prescription of steroid therapy [10] as well as of some disease modifying treatments is associated with subsequent risks of mood disturbance, such as depressive episodes [11], manic and psychosis [10, 12] the etiology and pathogenesis of these comorbidities are still poorly understood.

There are also indications that affective symptoms become apparent at an early stage of the disease, even before the definite diagnosis of MS [13–15], and remain stable for at least 3 years [16]. Support for this assumption comes from a recent retrospective cohort study in which an association between stress-related disorders and an increased risk of subsequent autoimmune diseases was shown [17]. The association between autoimmune disease and affective disorders also indicated an immunological contribution to the development of affective symptoms [18] and hence affective symptoms may comprise the first manifestations of MS [15]. From this, it can be inferred that the presence of neuropsychiatric comorbidities indicates a clear need for intervention as early as possible [14, 19].

Nevertheless, there is evidence suggesting that affective symptoms are still underdiagnosed and undertreated in the clinical standard care of patients with MS [20, 21]. Against this background, in this narrative review, we provide an overview of the incidence and prevalence of affective disorders and symptoms in MS and their impact on quality of life, as well as the current clinical assessment options.

## Methods

Included in this narrative review were studies with findings on the appearance and epidemiology of affective symptoms and disorders which include depression, anxiety, bipolar disorder, euphoria, and pseudobulbar affect (pathological laughing and crying) in patients with MS. Furthermore, studies focusing on the association between the mentioned affective symptoms or disorders and quality of life were considered, and the findings from the available diagnostic assessment tools are discussed. The literature search was limited to studies that were published between 2009 and 2021, even though a few selective earlier studies were also included if they showed basic insights concerning the covered domains. Case reports were not included. The articles of interest were identified in the ISI Web of Science, ScienceDirect, PubMed, EBSCO Psychology (PsycINFO, PsycARTICLES, PSYNDEXplus), Google Scholar, and Cochrane library.

## Results

The 65 analyzed articles for this literature review are displayed in the appendix (Tables 2, 3, 4, 5, 6, 7, 8, 9, 10, 11, 12, 13), sorted chronologically by year (and alphabetically by author name). The tables contain information about the first author, publication year, investigated symptom or disorder category, total sample size of MS population, age, gender, country of data, included diagnostic assessment tools, main findings, and comments.

### Depression

According to cross-national data, major depression represents one of the most common affective disorders with a lifetime prevalence of 20% in the general population [22]. In patients with MS, depression seems to occur more often [23–27] and there is evidence suggesting that patients with MS show a risk of up to 50% of developing major depression in their lifetime [28]. In comparison to the general population, patients with MS have been found to show an elevated point prevalence in depression by 9.3% [8] up to 23.7% [5]. Adjusting for age and sex, the incidence of depression was found to be 71% higher in a group of patients with MS than in a matched healthy control sample [6]. When considering self-reports only, depression rates were even higher. For example, 54% of the patients with MS in the United Kingdom were found to experience depression at some point in their life [29].

In the general population, depressive symptoms are reported to be more frequent in females (12.4%) than in males (6.1%) [8]. However, Théaudin and colleagues [30] did not find any evidence of sex differences concerning depression in MS patients.

Furthermore, compared to patients experiencing depressive symptoms without underlying medical illness, patients with MS showed a comparable clinical presentation of depressive symptomatology. The observed differences between depressive patients and patients with MS were found in specified symptoms: fatigue and irritability were pronounced in patients with MS, while anhedonia and loss of interest were pronounced in patients with a Major Depression [24, 31].

### Anxiety

Compared to the lifetime prevalence rates for depression, the reported prevalence rates of an anxiety disorder or anxiety symptoms in MS (general anxiety disorder, social anxiety, agoraphobia, etc.) are more homogeneous, ranging from 20 to 44.5% [32–36]. According to the World Health Organisation (WHO), 8.5% of people in the normal population experience a general anxiety disorder. In contrast, Poder and colleagues (2009) showed that 30.6% of patients with MS were diagnosed with social anxiety disorder, while half of these patients were also diagnosed with general anxiety [37]. Compared to the general population, patients with MS were found to show a higher point prevalence in generalized anxiety disorder by 2.2% [8]. The systematic review by Marrie and colleagues in 2015 also revealed an elevated average point prevalence for anxiety of 21.9% in the MS population [5]. An increased risk of anxiety was found in the pre- and post-diagnostic period of MS, compared to control groups [7, 38]. Female patients with MS and patients with a relapsing–remitting disease course and a worse degree of functional disability showed higher symptoms of anxiety [29, 30].

After adjusting for age and sex, the incidence of an anxiety disorder was still 42% higher in patients with MS than in a matched healthy control sample [6]. In a recent meta-analysis, Boeschoten and colleagues (2017) demonstrated that 22.1% of patients with MS were affected by anxiety [35]. As is the case for depression, when considering self-reports only, anxiety rates were higher as 46.9% of patients with MS in the United Kingdom indicated experiencing anxiety at some point in their life [29].

### Interaction of depression and anxiety

With respect to the interaction between depression and anxiety in patients with MS, the results are inconsistent. Some studies indicate that depression and anxiety may be interdependent [33, 39] while more recent findings show that non-somatic symptoms of depression (excessive worry, fear of losing control, inability to relax, etc.) and employment status were risk factors for higher levels of anxiety symptoms in MS [40]. Askari et al. (2014) concluded that depression and the disability level are independent predictors of anxiety [32]. Other studies revealed that the relationship between different aspects of depression and anxiety might change over the course of MS, such that patients may report a stronger link between somatic symptoms of depression with symptoms of anxiety at later disease stages than at earlier stages [40]. Kehler and Hadjistavropoulos (2009) showed that patients with MS and depression had higher levels of health anxiety, the fear or worry about health, compared to an age-matched control group [41]. Nevertheless, although there are no clear correlations between anxious and depressive symptoms in patients with MS the suffering of pressure in the medical history of the affected patients is indisputable.

### Bipolar disorder

A further manifestation of affective disorder that should be considered in patients with MS is a bipolar spectrum disorder. Although findings vary from country to country, a large cross-sectional survey across 11 countries found the overall lifetime prevalence of bipolar spectrum disorder to be 2.4% in the general population [42]. There are currently only a few findings concerning the epidemiology, prevalence, and incidence of bipolar disorder in MS patients or correlations between MS and bipolar disorder [12]. Relative to age- and sex-matched controls, Carta and colleagues (2013) reported an odds ratio of 44.4 for experiencing a bipolar disorder together with MS [43]. Marrie et al. (2015) reported a lifetime prevalence of up to 16.2% in patients with MS. An increased lifetime prevalence of bipolar spectrum disorder (bipolar disorders type I and II and cyclothymic disorders) has also been reported for patients with MS when compared to age- and sex-matched controls [5]. After adjusting for age and sex, the incidence of bipolar disorder was 99% higher in MS patients than in the matched healthy population [7].

### Euphoria

Pathological euphoria as one component of the affective symptoms in MS was first described in the nineteenth century, whereby approximately 10% of the patients were diagnosed to be affected by the pathological expression of euphoria [44]. Given that there was no guidance for a common operationalization of euphoria in patients with MS, different subsequent definitions must be considered. Cottrell and Wilson (1926) provided a classical description of euphoria in which three associated but independent types of euphoria are distinguished [45, 46]: (1) “euphoria sclerotica”: a mental state or mood of cheerfulness and happiness; (2) “eutoniasclerotica”: a feeling of being physically well despite physical deficits; patients in this state are convinced that they can do anything and they are oblivious of their actual physical disability; and (3) “spessclerotica”: a great optimism concerning the future and the prospects of complete recovery from the symptoms [46]. Based on this definition of “euphoria sclerotica,” Paparrigopoulos et al. (2010) described euphoria as a fixed mental state of unusual cheerfulness and optimism about the future despite the presence of a neurological disability. The authors suggested that euphoria should be regarded as a personality change that should be considered distinct from hypomania despite having superficial similarities with it [26]. Duncan et al. (2015) put forward a contemporary definition of euphoria as an “overly optimistic” or “unrealistic optimism” [45]. Using the classical euphoria definition and interviewing method developed by Cottrell and Wilson (1926), Duncan et al. (2016) found high proportions of Cottrell and Wilson’s three types of euphoria (between 63 and 70%, depending on the type of euphoria) in patients with MS. In contrast, using a more contemporary definition and measurement instrument (questions of the Neuropsychiatric Inventory by Cummings (1997) [47]), only 11% of patients with MS were diagnosed as having symptoms of euphoria. The proportions of Cottrell and Wilson’s three types of euphoria in the control group varied between 86 and 94% and thus were significantly higher than the proportions found in the MS group. In contrast, when the Neuropsychiatric Inventory was used, the proportion of persons diagnosed with euphoria in the control group was 4% and thus was *lower* than the proportion found in the group of patients with MS although the relevant statistical test narrowly missed the conventional criterion of statistical significance [48]. Nevertheless, there are some clear correlations, and patients with symptoms of euphoria were more likely to suffer from a progressive disease form and showed more significant structural pathology [45].

### Pseudobulbar affect

A particular type of affective symptom that occurs in patients with MS is pseudobulbar affect. This affective disinhibition syndrome is also known as pathological laughing and crying and is characterized by spontaneous, involuntary, and uncontrollable outbursts of contextually inappropriate laughing or crying that are inconsistent with the patient’s underlying feelings or incongruent with external triggers [49]. Uncontrollable crying seems to be more common than laughing and this mood disturbance has been described in 10% of the MS patients [50]. There are no studies on the prevalence rate of pseudobulbar affect in the general population [49]. A recent study suggests that there may be a relationship between the occurrence of pseudobulbar affect and cognitive impairment in patients with MS. Specifically, deficits in processing speed, visuospatial memory, verbal learning, and fluency in patients with MS have been reported to be associated with pseudobulbar affect [51]. Although more scientific evidence is needed in this area, based on previous indications attention should also be given to pseudobulbar affect in the care of patients with MS.

### Association between affective symptoms and quality of life

Affective symptoms such as depression, anxiety, and bipolar disorder are associated with decreased adherence to MS treatment [52], more unfortunate disease progression, cognitive deficits [33], higher suicide risk [53], and reduced quality of life [3, 20, 27]. Depressive symptoms were found to covary with fatigue and reduced cognitive performance, especially concerning information processing speed, attention, working memory, and executive function [54]. Both symptoms of fatigue and reduced cognitive performance may severely affect patients’ quality of life.

Higher levels of health anxiety are associated with greater emotional preoccupation, more use of social support, and reduced use of adaptive coping strategies to manage disabilities in MS [41]. Recent findings show an association between emotional dysregulation and lower health-related quality of life. Maladaptive strategies and difficulties in regulating emotions mediated the associations of depression and anxiety with the quality of life in patients with a progressive course of MS [55].

There seem to be no studies on the possible interactions of euphoria or pseudobulbar affect with important aspects of quality of life such as physical disability or cognitive impairment. The most elaborated studies on quality of life in patients with MS with affective symptoms are available for depression.

Early studies seemed to indicate that greater disease severity and shorter disease duration may be associated with a clinically significant level of depressive symptoms in patients with MS [56]. However, there are also controversial findings concerning the correlation between depression and physical limitations in patients with MS: previous findings provided no evidence of a direct relationship between depression and the Expanded Disability Status Scale [57] as a measure of physical disability in MS [58]. More recent findings showed that patients with MS and comorbid depression had a significantly increased risk of worsening disability [59]. The presence of depression, anxiety, or bipolar disorder was associated with a higher EDSS score in female patients with MS (adjusted for disease duration and progression, age, sex, socioeconomic status, physical comorbidity count, and disease-modifying therapy exposure) [60]. Furthermore, Honarmand et al. (2011) reported a strong association between unemployment and the severity of depression in MS. This work highlighted the need to consider more than physical functioning for the prediction of the employment status as an essential aspect of quality of life [61].

### Diagnostic assessment tools

Although there are a few recommendations in the research to perform routine screenings for diagnosable affective disorder in MS, clinicians generally collect information about affect symptoms through unstructured and semi-structured interviews [62–64], and a clear diagnostic procedure is still lacking [21, 35].

Specifically, affective symptoms deserve more attention in patients with MS given that they have a substantial impact on disease progression and quality of life [27]. Mood disturbances may reduce the ability to cope with disabilities in MS [39, 41], cognitive performance [33, 54], and adherence to therapy [52]. However, it is important to note that despite the apparent plausibility, causal interpretations of the typically correlational findings in this domain are not warranted.

An overview of the diagnostic methods used for diagnosing affective disorders and symptoms in MS is provided in Table 1. In clinical contexts, a semi-structured interview is applied to identify DSM-IV diagnoses (SCID) including anxiety and depression [65]. However, this method is time-consuming and thus is seldom used in clinical practice for the care of patients with MS. Typically, self-reporting measures are used instead and a systematic review revealed the need to assess the utility of these measures [66]. The following screening measures for depression were frequently reported: the Beck Depression Inventory-II (BDI-II) [67], the Center for Epidemiologic Studies Depression rating scale (CES-D) [68], and the Chicago Multiscale Depression Inventory (CMDI) [69]. Regarding screening measures for anxiety, the following self-reporting measures are partially validated and usually used in MS: the Beck Anxiety Inventory (BAI) [32], and the Anxiety 7-item and Generalized Anxiety Disorder Scale (GAD-7) [70]. Some measures assessing both depression and anxiety that are used include the 9-item Patient Health Questionnaire (PHQ-9) [71], the Hospital Anxiety and Depression Scale (HADS) [68, 72], and the Patient-Reported Outcome Measurement Information System (PROMIS) [66]. A systematic review confirmed the need for early intervention and treatment of anxiety throughout the course of MS [73]. Therefore, research has already addressed the need for improvement in diagnosing depression [74, 75] and anxiety [70, 72, 75] in MS. The Hospital Anxiety and Depression Scale has become a widely used screening instrument in patients with MS and it can be considered a valid instrument for assessing symptoms of both depression and anxiety [76].

**Table 1:** Diagnostic assessment tools for the listed affective burdens or mood disturbances

Diagnostic assessment tools	Depression	Anxiety	Bipolar disorder	Euphoria	Pseudobulbar Affect
SCID	x	x	–	–	–
BDI-II	x	–	–	–	–
CES-D	x	–	–	–	–
CMDI	x	–	–	–	–
BAI	–	x	–	–	–
GAD-7	–	x	–	–	–
PHQ-9	x	–	–	–	–
HADS	x	x	–	–	–
PROMIS	x	x	–	–	–
Mood Disorder Questionnaire	–	–	x	–	–
CNS-LS	–	–	–	–	x

*PCL* pathological laughing and crying, *SCID* Structured Clinical Interview for DSM Disorders, *BDI-II* Beck Depression Inventory-II, *CES-D* Center for Epidemiologic Studies Depression rating scale, *CMDI* Chicago Multiscale Depression Inventory, *BAI* Beck Anxiety Inventory, *GAD-7* Anxiety 7-item and Generalized Anxiety Disorder Scale, *PHQ-9* 9-item Patient Health Questionnaire, *HADS* Hospital Anxiety and Depression Scale, *PROMIS* Patient-Reported Outcome Measurement Information System, *CNS-LS* Center for Neurologic Study-Lability Scale

A more specialized instrument for manic and hypomanic symptoms is the Mood Disorder Questionnaire, which is a standardized screening questionnaire [77]. It is the most widely studied screening instrument for bipolar disorder [77] and has been applied in patients with MS [78]. Unfortunately, both the sensitivity and specificity of this instrument are low [77].

To detect and measure the severity of a pseudobulbar affect, the self-reporting method known as the Center for Neurologic Study-Liability Scale (CNS-LS) includes two subscales measuring labile laughter and tearfulness [12]. However, the CNS-LS was not developed for use in MS and a disease-related diagnostic procedure for euphoria does not seem to exist.

A considerable number of studies have revealed that the instruments to measure diagnosable affective symptoms which have been validated in patients with MS show similar characteristics and correlate with each other [65, 79] whereby there was no clear superiority of one instrument over the others. The instruments shared high negative predictive values; therefore, clinicians could exclude the presence of a definite diagnosis of depression or generalized anxiety disorder, although the diagnostic accuracy was low. Consequently, individuals with scores that are elevated but still below the cut-off points may experience subsyndromal symptoms, which are not discovered but also warrant clinical attention. Furthermore, a recent study in this context showed that less obvious symptoms such as social and emotional health problems can be more relevant in identifying mood disturbances in patients with MS than anxiety and depression [80]. This underpins the role of subsyndromal affective symptoms and the need for the identification of individuals with symptoms below the cut-off points of definite diagnostic criteria in the context of MS.

## Discussion and conclusion

This narrative review shows a large range of prevalence and incidence rates of affective symptoms in MS such as depression, anxiety, bipolar disorder, euphoria, and pseudobulbar affect. Focusing on individual studies could thus easily lead to an underestimation of the rates at which these affective symptoms occur [21].

One reason for the wide range in prevalence and incidence rates of affective symptoms in MS may be the use of widely varying diagnostic methods. However, even the lower ends of the reported prevalence and incidence rates suggest that a substantial proportion of patients with MS experience affective disorders and symptoms. Affective symptoms may even be the first manifestation of the disease [14, 15, 19]. Although pathological mood expressions such as euphoria and pseudobulbar affect were already identified in the first description of MS [44], investigations concerning symptoms that do not meet the criteria for a diagnosable disorder are still rare.

In addition to a few recommendations to screen patients with MS for definite psychiatric disorders [62–64, 81], there are no recommendations concerning how to proceed if symptoms fail to meet diagnostic criteria but are nevertheless a significant source of distress [63]. Such subsyndromal affective symptoms are reported spontaneously by individuals or collected in response to the demand of the clinicians (through interviews, questionnaires, checklists, or severity rating scales) but do not meet the diagnostic criteria (e.g., duration, intensity, and impact of functioning) of definite psychiatric diagnoses [63]. Most of the assessments used focused on single symptoms, which have been partially validated for use in MS patients but not specifically developed for this purpose. A first indication of how relevant subsyndromal symptoms are in dealing with emotional states was derived from a recent study which showed that symptoms such as social and emotional health problems in MS that are less obvious than anxiety and depression are important for the mental health of patients with MS [77]. Neglecting these symptoms would be problematic given that they are negatively associated with the quality of life of MS patients [18, 25, 78]. These deficiencies point to the need for an MS-related assessment tool to also detect subsyndromal affective symptoms in the clinical care of patients with MS.

The lack of a common procedure and sensitive assessment tool for less obvious or subsyndromal affective symptoms can partly explain the reason why the occurrence of affective symptoms is still underestimated and as a result, often remain untreated [21, 65]. Future studies are needed to facilitate better insights into the interaction of these symptoms and the underlying pathophysiology [50]. The elaboration of a common definition of relevant MS-related affective symptoms and mood disturbances, including relevant subsyndromal symptoms, should be focused on in future investigations as a deeper understanding of these issues could improve the care of MS patients. Translating these findings into a definition of a standardized diagnostic procedure is necessary for integrated treatment methods, including essentially the aspects of mental health.

## Appendix: Tables with included and reviewed research articles

Tables 2, 3, 4, 5, 6, 7, 8, 9, 10, 11, 12, 13.

**Table 2:** Included references

Author, Year	Symptom or disorder category	MS population	Diagnostic assessment tools	Main findings	Comments
Brown et al. 2009 [39]^a^	Depression, anxiety	*n* = 101 Age: 42.6 ± 10.7 Female (*n*): 81.0 Australia	BDI-II, STAI, SEFCI, LEDS, LOT; LOC, SSQ, 2- question of Pittsburgh sleep quality index	Depression predicted anxiety and anxiety predicted later depression. Psychological distress (i.e. anxiety, depression) was predicted by a combination of unhealthy behaviours (e.g. drug use, smoking, no exercise, or relaxation) and psychological factors (e.g. low optimism, avoidance coping), similar to the results of community-based studies. However, state-anxiety was also predicted by immunotherapy status	A 2-year prospective longitudinal study. Additional questionnaires: Coping questionnaire (66-item), a short screening battery (14-items) assessing life-style factors
Haussleiter et al. 2009 [50]^b^	Depression, anxiety, pseudobulbar affect, bipolar disorder, euphoria	NA	NA	In addition to the psychiatric manifestations of MS, many patients have reactive psychological problems that are often hard to distinguish from the ‘organic’ causation of psychopathology. In any event, psychiatric comorbidity in MS deserves greater clinical attention than has been previously paid, because the presence of psychopathology may have deleterious effects on the disease process and impair coping with disability	Information about prevalences and correlations of a wide range of psychiatric disorders
Honarmand et al. 2009 [75]^a^	Depression, anxiety	*n* = 140 Age: 44.6 ± 10.3 Female (*n*): 135 Canada	HADS, SCID-IV	A threshold score of 8 or greater on the HADS depression subscale provides a sensitivity of 90% and specificity of 87.3% (ROC area under the curve 0.938). The same cut-off score gives a sensitivity of 88.5% and a specificity of 80.7% on the anxiety subscale (ROC area under the curve 0.913), but for generalized anxiety disorder only. HADS is a useful marker of major depression and generalized anxiety disorder, but not other anxiety disorders, in MS patients	Self-report rating scales represent a challenge because of somatic confounders
Kehler et al. 2009 [41]^a^	Anxiety, QoL	*n* = 246 Age: 41.8 ± 10.2 Female (*n*): 200 Canada	SHAI, HADS, GNDS, WOCQ, CHIP	Participants with MS reported higher levels of health anxiety compared to an age-matched control sample. Compared to normal levels of health anxiety, participants with MS with elevated health anxiety were differentiated by endorsement of greater use of Emotional Preoccupation and Social Support and less use of Problem-Focused Coping. Participants with elevated health anxiety experienced greater disability and generalized anxiety. Health anxiety and generalized anxiety were both uniquely associated with Emotional Preoccupation coping, whereas only health anxiety was uniquely associated with Problem-Focused coping	Clinicians should be aware that health anxiety is elevated in people with MS, and that as many as 25% of individuals with MS report health anxiety in a clinical range
Marrie et al. 2009 [21]^a^	Depression, anxiety, bipolar disorder	*n* = 8983 Age: 52.7 ± 10.4 Female (%):75.8 North America	CESD	Mental comorbidity affected 4264 (48%) responders; depression most frequently (4012, 46%). Among participants not reporting mental comorbidity, 751 (16.2%) had CESD scores ≥ 21 suggesting undiagnosed depression. Lower socioeconomic status was associated with increased odds of depression, undiagnosed depression, and untreated depression. Mental comorbidity remains underdiagnosed and undertreated in MS. Patients of lower socioeconomic status bear a disproportionate share of the burden of depression	Participants in the North American Research Committee on MS

The selected studies are sorted chronologically by year and alphabetically by author name. Types of articles: ^a^Empirical article; ^b^Review article; Definition papers were not included in the table; *n* = sample size; age: mean ± standard deviation; NA = Not applicable/reported

Abbreviations: *BDI-I/-II* Beck Depression Inventory, *CES-D (-10)* Center for Epidemiologic Studies Depression Scale (10-item scale, identical to the CES-D), *CHIP* Coping with health, injuries, and problems scale, *GNDS* Guy’s neurological disability scale, *HADS (-A/-D)* Hospital Anxiety and Depression Scale (-Anxiety Scale/-Depression Scale), *LEDS* life-events and difficulties schedule, *LOT* life orientation test (revised), *LOC* health locus of control scale, *MS* Multiple Sclerosis, *QoL* quality of life, *SCID (-IV)* Structured Clinical Interview for the (for DSM-IV), *SHAI* Short Health Anxiety Inventory, *SSQ* Sarason social support questionnaire, *STAI* State-Trait Anxiety Inventory, *WOCQ* Ways of coping questionnaire

**Table 3:** Included references

Author, year	Symptom or disorder category	MS population	Diagnostic assessment tools	Main findings	Comments
Poder et al. (2009) [37]^a^	Anxiety, QoL	*n* = 245 Age: 46.1 ± 10.6 Female (*n*): 200 Canada	HADS, DMSRU, HRQoL, CAGE, HUI, SCID, SPIN	30.6% of the sample had clinically significant social anxiety symptoms as defined by a SPIN threshold of 19. Neurological disability did not differ between these groups. Half of those with social anxiety also had general anxiety and a quarter had depression. The severity of social anxiety symptoms was not related to the level of neurological disability, although it was associated with reduced health related QoL. Clinicians should consider screening for symptoms of social anxiety, especially as MS severity or duration are not good indicators of its likely presence or severity	Optimal screening methods, and potential treatments of social anxiety symptoms in MS patients is warranted
Paparrigopoulos et al. 2010 [26]^b^	Depression, anxiety, euphoria, pathological laughing and crying	NA	NA	Neuropsychiatric symptoms are present in most patients with MS even in the early stages of the disease. Major depression is the most common neuropsychiatric disorder in MS and a key determinant of morbidity, mortality, patient QoL and possibly disease progression; it has also been found to correlate with the caregiver’s distress and QoL. Early recognition and effective management of depression and other neuropsychiatric symptoms are essential parts of optimal care for patients with MS.	A comprehensive biopsychosocial neuropsychiatric, and not strictly neurological, approach is warranted in the diagnostic and treatment modalities provided to patients with MS
Da Silva et al. 2011 [56]^a^	Depression, anxiety, QoL	*n* = 325 Age: 39.5 ± 10.8 Female (*n*): 205 Portugal	HADS, MSSS	The study results confirmed the high prevalence of anxiety and depression in MS patients and significant associations between depression scores and physical manifestations of MS. Age, disease duration, age at onset, EDSS, and MSSS were positively associated with depression scores. Levels of anxiety and depression were significantly higher for MS patients’ group than healthy subjects. Age, Low education in MS was significantly associated with more anxiety and depression symptoms	The study findings support a close linkage between depressive mood and physical manifestations of MS
Honarmand et al. 2011 [61]^a^	Depression, QoL	*n* = 106 Age: 44.7 ± 8.3 Female (*n*): 82 Canada	MSFC, BRBN, HADS, NEO-FFPI	The findings highlight the utility of the MSFC as a predictor of unemployment in MS. Furthermore, a strong association was found between unemployment and the personality construct ‘‘Agreeableness’’, and severity of depression	Focus on unemployment in MS patients, as an important part of QoL
Iacovides et al. 2011 [49]^b^	Bipolar disorder, euphoria, pseudobulbar affect, pathological laughing and crying, QoL	NA	NA	Psychopathological symptoms and signs should also be considered in the differential diagnosis in the first clinical manifestations of MS – even in the absence of neurological clinical findings, or in the presence of ‘mild’ ones Even though the higher prevalence of psychiatric disorders in MS is well established, such disorders remain underdiagnosed and undertreated. A shift in the clinical suspicion towards the psychiatric morbidity in MS patients and the optimal treatment of those disorders is fundamental	Psychiatric disorders and symptoms such as bipolar disorder, pseudobulbar affect, euphoria sclerotica, anxiety and personality changes are also reported to be overrepresented in MS patients

The selected studies are sorted chronologically by year and alphabetically by author name. Types of articles: ^a^Empirical article; ^b^Review article; Definition papers were not included in the table. *n* = sample size; age: mean ± standard deviation; NA = Not applicable/reported

Abbreviations: *BRBN* Brief Repeatable Battery of Neuropsychological Tests, *CAGE* Questions Adapted to Include Drug Use, *DMSRU* Dalhousie MS Research Unit, *HADS (-A/-D)* Hospital Anxiety and Depression Scale (-Anxiety Scale/-Depression Scale), *HRQoL* Health-Related QoL, *HUI* Health Utilities Index, *MS* Multiple Sclerosis, *MSFC* MS Functional Composite, *MSSS* MS Severity Scale, *NEO-FFPI* Five-Factor Personality Inventory, *NPI* Neuropsychiatric Inventory, *QoL* Quality of life, *SCID (-IV)* Structured Clinical Interview for the (for DSM-IV), *SPIN* Social Phobia Inventory

**Table 4:** Included references

Author, year	Symptom or disorder category	MS population	Diagnostic assessment tools	Main findings	Comments
Tarrants et al. 2011 [52]^a^	Depression, QoL	*n* = 436 Age: NA Female (*n*): 381 USA	ICD-9-CM code	MS patients with comorbid depression were approximately half as likely to be adherent to their DMT relative to patients with MS without depression. Although treatment with antidepressant therapy generally did not improve the likelihood of adherence, treatment with antidepressants for at least 6 months was associated with better adherence to DMT	None
Zimmerman et al. 2011 [77]^b^	Bipolar disorder	NA	MDQ, DSM-IV, CIDI, SCID, MINI	Across all studies the sensitivity of the MDQ was 61.3%; specificity, 87.5%; positive predictive value, 58.0%; and negative predictive value, 88.9%. Compared to the studies using the MDQ for psychiatric outpatients, studies using it in the general population found it to have much lower sensitivity and positive predictive value, and higher specificity and negative predictive value. The MDQ’s sensitivity was higher in detecting bipolar I disorder than bipolar II disorder (66.3% vs. 38.6%). Lowering the threshold to identify cases markedly improved the MDQ’s sensitivity, with only a modest reduction in specificity	Studies of the best symptom cutoff to identify cases have produced inconsistent findings. Based on current available evidence, routine clinical use of the MDQ cannot be recommended because of the absence of studies simultaneously examining both the potential benefits and costs of screening
Jones et al. 2012 [29]^a^	Depression, anxiety	*n* = 7786 Age: 50.9 ± 11.5 Female (*n*): 3253 United Kingdom	HADS	Anxiety and depression rates were notably high, with over half (54.1%) scoring ≥ 8 for anxiety and 46.9% scoring ≥ 8 for depression. Women with relapsing–remitting MS were more anxious than men with this type, and then women with other types of MS. Within each gender, men and women with secondary progressive MS were more depressed than men or women with other types of MS	Anxiety and depression are highly prevalent in people with MS, indicating that their mental health needs could be better addressed to provide the best care for people with MS
Leonavičius et al. 2012 [2]^a^	Depression, QoL	*n* = 270 Age: 42.4 ± 11.7 Female (*n*): 187 Lithuania	ICD-10	Depression was present in 20.7% of patients, with a similar prevalence between the sexes. Patients who indicated that MS interfered with their family life were at significantly increased risk of depression. Patients who identified the need to pay more attention to MS, and to include more medications in reimbursement lists, as factors that would improve the management of MS, were more likely to be diagnosed with depression	The negative impact of MS on family life is an important factor contributing to the risk of depression
Wood et al. 2012 [36]^a^	Anxiety, depression	*n* = 198 Age: 48.2 ± 11.4 Female (*n*): 137 Australia	HADS, FSS	Prevalence of anxiety was 44.5%, depression 18.5%, and fatigue 53.7%. Prevalence of anxiety (but not depression or fatigue) decreased by 8.1% per year of cohort observation, with the effect more pronounced in women (14.6%) than men (2.6%). There was no apparent seasonal variation in the prevalence of any of the three factors	All three factors (anxiety, depression and fatigue) occurred contemporaneously at cohort entry in a higher proportion of the cohort than expected by chance
Carta et al. 2013 [43]^a^	Depression, bipolar disorder	*n* = 201 Age: 38.9 ± 10.0 Female (*n*): 140 Italy	DSM-IV, ANTAS-SCID, MDQ, SF-12	Compared to controls, MS patients had a higher lifetime prevalence of DSM-IV. Major depressive disorder; Bipolar disorder I, Bipolar disorder II and Cyclothymia. As people with MS had a higher risk of depressive and bipolar spectrum disorders, ratio MDD/bipolar spectrum disorders was lower among cases indicating a higher association with Bipolar Spectrum Disorders and MS	The results suggest a risk of under-diagnosis of Bipolar disorder in MS and caution in prescribing Adjustment disorders to people with depressive episodes in MS without prior excluding Bipolar disorder

The selected studies are sorted chronologically by year and alphabetically by author name. Types of articles: ^a^Empirical article; ^b^Review article; Definition papers were not included in the table. *n* = sample size; age: mean ± standard deviation; NA = Not applicable/reported

Abbreviations: *ANTAS* Advanced Neuropsychiatric Tools and Assessment Schedule, *CIDI* Composite International Diagnostic Interview, *CIS* Clinical Interview Schedule, *DMT* Disease-modifying therapy, *DSM-IV* Diagnostic and Statistical Manual of Mental Disorders, 4th ed, *FSS* Fatigue Severity Scale, *HADS (-A/-D)* Hospital Anxiety and Depression Scale (-Anxiety Scale/-Depression Scale), *ICD-9/-10* International Classification of Disease, *MDQ* Mood Disorders Questionnaire, *MINI* Mini–International Neuropsychiatric Interview, *MS* Multiple Sclerosis, *QoL* quality of life, *SCID (-IV)* Structured Clinical Interview for the (for DSM-IV), *SF-12* Short-Form-Health Survey

**Table 5:** Included references

Author, year	Symptom or disorder category	MS population	Diagnostic assessment tools	Main findings	Comments
Leonavičius et al. 2013 [34]^a^	Anxiety	*n* = 312 Age: 42.0 ± 12.5 Female (*n*):196 Lithuania	HADS, ICD-10	The results show a significant level of anxiety and low level of social activity in people with MS. Anxiety in MS patients was strongly related with younger age, shorter MS duration, prevalence of depression and lower level of social activity. A higher level of social activity was significantly related with older urban MS patients who indicated family status as living together and longer MS duration.’	Patients were asked ‘Did you take an active part during the last year in MS group activities such as clubs, meetings, camps, trainings, self-help groups, or other projects?
Marrie et al. 2013 [7]^a^	Anxiety, bipolar disorder	*n* = 4192 Age at symptom onset: 33.2 ± 11.1 Female (%):71.7 Canada	ICD-9-/10	Compared to medical records, administrative definitions showed moderate agreement for any mental comorbidity, mood disorders and depression, fair agreement for anxiety and bipolar disorder, and near perfect agreement for schizophrenia. The age-standardized prevalence of all mental comorbidities was higher in the MS than in the general populations	The prevalence of mental comorbidities is increased in MS compared to the general population
Thielscher et al. 2013 [62]^a^	Depression	*n* = 5137 Age: 46.1 ± 18.9 Female (%):70.9 Germany	ICD-10	Alzheimer’s/dementia, MS, and Parkinson’s are riskier than epilepsy in terms of the likelihood of the development of depression. In patients with MS, 35% developed depression. Earlier estimates found a risk of up to 50–56%; and that the risk was higher in younger, single, better educated, and unemployed patients. It is not yet clear whether the depression is pathophysiological or reactive in nature	Analysis of how many patients’ developed depression within five years
Askari et al. 2014 [32]^a^	Depression, anxiety	*n* = 180 Age: 32.4 ± 8.7 Female (*n*): 151 Iran	BDI, BAI	Mean BDI and BAI were not statistically different between male and female participants. Patients with higher levels of disability (higher EDSS) had significant higher BDI and BAI scores and there were significant positive correlations between EDSS and BDI and BAI scores. Patients with SPMS had significantly higher BDI and BAI scores	Multiple linear regression analysis showed that depression and disability level were independent predictors of anxiety in patients
Caceres et al. 2014 [16]^a^	Depression, euphoria	*n* = 110 Age: 36.6 ± 10.6 Female (%): 67.3 Latin America	BDI-II, NPI, MSNQ, Zarit's Caregiver's Burden Scale	In addition to overall neurological disability, both cognition and neuropsychiatric symptoms distinguished patients and controls. The prevalence of cognitive impairment was 34.5% and 20.9% presented with clinically significant neuropsychiatric symptomatology. Cognitive impairment was a significant predictor of employment status	In addition, cognitive status was assessed
Carta et al. 2014 [78]^a^	Depression, bipolar disorder, QoL	*n* = 201 Age: 38.9 ± 10.0 Female (*n*): 140 Italy	DSM-IV, ANTAS-SCID, MDQ, SF-12	MS was the strongest determinant in worsening the QoL in the overall sample. Both MDD and BD type-II lifetime diagnoses were significantly associated with a poorer QoL in the total sample as in cases of MS. In MS the impairment of the QoL attributable to BD type-II was even greater than that in MDD.MDD as well BD type-II are co-determinants in worsening QoL in MS	Clinicians should consider depressive symptoms as well as the hypomanic and mixed components in MS
Feinstein, Magalhaes et al. 2014 [27]^b^	Depression, QoL	NA	DSM-III/-IV, BDI/-II, HDRS, ZSRDS, CMDI, HAM-A, CES-D	Depression is more common in patients with MS than in the general population, and substantially impairs QoL; suicide rates are also elevated in patients with MS. To advance research and clinical practice, depression in patients with MS will require a rigorous definition based on quantitative assessment. Structural brain changes on MRI account for almost 50% of the variance in the presence of MS-related depression; genetic, biochemical, immunological, and psychosocial factors have also been implicated	Antidepressants are modestly effective, but adverse effects can preclude adequate dosing; cognitive–behavioural therapy is also effective

The selected studies are sorted chronologically by year and alphabetically by author name. Types of articles: ^a^ Empirical article; ^b^ Review article; Definition papers were not included in the table. *n* = sample size; age: mean ± standard deviation; NA = Not applicable/reported

Abbreviations: *ANTAS* Advanced Neuropsychiatric Tools and Assessment Schedule, *BAI* Beck Anxiety Inventory, *BDI (-I/-II)* Beck Depression Inventory, *BD I/II* Type I/II of Bipolar Disorder, *CES-D (-10)* Center for Epidemiologic Studies Depression Scale (10-item scale, identical to the CES-D), *CMDI* Chicago Multiscale Depression Inventory, *DSM(-III/-IV)* Diagnostic and Statistical Manual of Mental Disorders, *HADS (-A/-D)* Hospital Anxiety and Depression Scale (-Anxiety Scale/-Depression Scale), *HAM-A* Hamilton Rating Scale for Anxiety, *HDRS* Hamilton Depression Rating Scale, *ICD-9/-10* International Classification of Disease, *MDD* Major Depressive Disorder, *MDQ* Mood Disorders Questionnaire, *MS* Multiple Sclerosis, *QoL* Quality of life, *SF-12* Short-Form-Health Survey, *SPMS* Secondary progressive MS, *ZSRDS* Zung Self-Report Depression Scale

**Table 6:** Included references

Author, year	Symptom or disorder category	MS population	Diagnostic assessment tools	Main findings	Comments
Fragoso et al. 2014 [64]^b^	Depression, anxiety	NA	DSM-IV	There are few clinical trials on treating depression in MS and no agreed recommendations for its assessment and follow-up. We present evidence-based recommendations for several aspects of depression in MS, including screening for depression, recognition of other concomitant psychiatric conditions, suicide risk, disability, fatigue, cognition, adherence to treatment, the effect of drugs used to treat MS on depression and possible pharmacological treatments for depression in MS Clinicians should be aware that patients with MS have greater risk of anxiety, bipolarity and obsessive- compulsive behaviour	Patients with MS who already have depression may also develop other less common, but equally distressing psychiatric symptoms
Goretti et al. 2014 [33]^a^	Anxiety, QoL	*n* = 190 Age: 37.5 ± 9.9 Female (*n*): 140 Italy	BRB, STAI, BDI, FSS	State anxiety was related to failure on the SDMT, and marginally, to failure on the PASAT-3, and to the presence of cognitive impairment. Moderate/severe depression was detected in 38 (20%) patients and fatigue in 109 (57%). Higher depression scores were related to impairment on the Stroop Test	Association of anxiety and depression with cognitive function
Minden et al. 2014 [63]^b^	Pseudobulbar affect, QoL	NA	HADS, BDI-I/-II, CES-D, CMDI, CNS-LS, CIDI, DIS, DSM-IV, GHQ, HDRS, HAM-A, HCL-20, MSIS-29, MSQLI, PANAS, PHQ-9, PSE, POMS, SADS, SCID-IV, SWLS, STAI, 1-/2- question screen	Clinicians may consider using the CNS-LS to screen for pseudobulbar affect, the BDI and a 2-question tool to screen for depressive disorders and the GHQ to screen for broadly defined emotional disturbances. Evidence is insufficient: 1. to support the use of other screening tools, the possibility that somatic/neurovegetative symptoms affect these tools’ accuracy, or the use of diagnostic instruments or clinical evaluation procedures for identifying psychiatric disorders in MS. 2. to support/refute the use of antidepressants and individual and group therapies reviewed herein. For pseudobulbar affect, a combination of dextromethorphan and quinidine may be considered. 3. to determine the psychiatric effects in individuals with MS of disease-modifying and symptomatic therapies and corticosteroids; risk factors for suicide; and treatment of psychotic disorders	Research is needed on the effectiveness in individuals with MS of pharmacological and non-pharmacological treatments frequently used in the non-MS population
Mrabet et al. 2014 [58]^a^	Depression, QoL	*n* = 50 Age: 20–50 Female (*n*): 45 Tunisia	GDS	Association between MS and depression is common and known. Prevalence of depression was 65%. Coexistence of other psychiatric disorders was found in around 10% of patients. Mood disorder was inaugural in some cases and delayed in others. This suggests that depression in MS may be linked to the disease or a result of a functional disability process. Occurrence of depression was not directly related to disease severity in all cases studied	Depression is a possible manifestation of MS. This mood disorder is due to the demyelinating brain damage or to a genetic susceptibility. However, a fortuitous association cannot be excluded

The selected studies are sorted chronologically by year and alphabetically by author name. Types of articles: Types of articles: ^a^Empirical article; ^b^Review article; Definition papers were not included in the table. *n* = sample size; age: mean ± standard deviation; NA = Not applicable/reported

Abbreviations: *BDI (-I/-II)* Beck Depression Inventory, *CES-D (-10)* Center for Epidemiologic Studies Depression Scale (10-item scale, identical to the CES-D), *CIDI* Composite International Diagnostic Interview, *CIS* Clinical Interview Schedule, *CMDI* Chicago Multiscale Depression Inventory, *CNS-LS* Center for Neurologic Study Emotional Lability Scale, *DIS* Diagnostic Interview Schedule, *DSM(-III/-IV)* Diagnostic and Statistical Manual of Mental Disorders, *FSS* Fatigue Severity Scale, *GDS* Geriatric Depression Scale, *GHQ (-28/-30)* General Health Questionnaire, *HADS (-A/-D)* Hospital Anxiety and Depression Scale (-Anxiety Scale/-Depression Scale), *HAM-A* Hamilton Rating Scale for Anxiety, *HCL-20* Hopkins Symptom Checklist, *HDRS* Hamilton Depression Rating Scale, *MS* Multiple Sclerosis, *MSIS-29* MS Impact Scale, *MSQLI* MS QoL-Inventory, *PANAS* Positive and Negative Affect Scale, *PHQ-2/-9* Patient Health Questionnaire, *POMS* Profile of Mood States, *PROMIS-D-8* Patient Reported Outcome Measurement Information System Depression 8-item bank, *PSE* Present State Examination, *QoL* Quality of life, *SADS* Schedule for Affective Disorders and Schizophrenia, *SCID (-IV)* Structured Clinical Interview for the (for DSM-IV), *STAI* State-Trait Anxiety Inventory, *SWLS* Satisfaction with Life Scale

**Table 7:** Included references

Author, year	Symptom or disorder category	MS population	Diagnostic assessment tools	Main findings	Comments
Simpson et al. 2014 [23]^a^	Depression, anxiety, bipolar disorder	*n* = 3826 Age: 53.4 ± 12.8 Female (*n*): 2767 Scotland, United Kingdom	NA	People with MS had a higher prevalence for mental conditions examined after adjustment for age, sex and socioeconomic status; being highest for depression, followed by anxiety, and problematic drug use. A diagnosis of an eating disorder was more prevalent, but the numbers within the eating disorder group were very small (n = 31). There was no statistically significant difference in prevalence of schizophrenia/bipolar disorder or learning disability, whilst dementia, and alcohol misuse were significantly less common amongst people with MS	Physical and mental health comorbidity is common in people with MS: nationally representative cross-sectional population database analysis
Terrill et al. 2014 [70]^a^	Anxiety	*n* = 513 Age: 51.4 ± 10.9 Female (*n*): 419 USA	HADS-A, PHQ-9 GAD-7	Findings support the reliability and internal validity of the GAD-7 for use in MS. Correlational analyses revealed important relationships with demographics, disease course, and depressive symptoms, which suggest the need for further anxiety research. Higher GAD-7 scores were observed in women and individuals with secondary progressive MS. Individuals with higher GAD-7 scores also endorsed more depressive symptoms	None
Watson et al. 2014 [72]^a^	Depression, anxiety	*n* = 34 Age: 48.5 ± 11.1 Female (*n*): 24 United Kingdom	BAI, BDI-II, HADS, SCAN, ICD-10, DSM- IV	The BDI-II and HADS can be used to identify mood disorders in people with MS. The BDI-II and HADS were both found to be valid measures to detect depression and anxiety in people with MS. An optimum cutoff score of 23 for the BDI-II yielded high sensitivity (85%) and high specificity (76%). An optimum cutoff score of 11 for the HADS demonstrated high sensitivity and specificity for both the Anxiety subscale (sensitivity 90%, specificity 92%) and the Depression subscale (sensitivity 77%, specificity 81%). The BAI had high sensitivity (80%) but poor specificity (46%) for detecting anxiety	Although the measures used in this study demonstrated high sensitivity and specificity, they were not perfect, and some people were misclassified
Fischer et al. 2015 [74]^a^	Depression	*n* = 31 Age: 49.1 ± 1.9 Female (*n*): 17 Germany	MINI, DSM-IV, BDI, IDS-SR_30_	BDI and IDS-SR_30_ total score were significantly correlated. The IDS-SR_30_ total score, cognitive subscore, and BDI showed excellent to good accuracy. Both the IDS-SR_30_ and the BDI are useful to quantify depressive symptoms showing good sensitivity and specificity. The IDS-SR_30_ cognitive subscale may be useful as a screening tool and to quantify affective/cognitive depressive symptomatology	Evaluation of diagnostic accuracy for major depression in MS using self-report questionnaires
Hartoonian et al. 2015 [40]^a^	Depression, anxiety	*n* = 513 Age: 51.4 ± 10–9 Female (*n*): 417 USA	NRS, FSS, HADS–A	After adjusting for demographic and disease related variables, anxiety, employment, and non-somatic depressive symptoms at baseline significantly predicted anxiety at Time 2. Interactions revealed significant effects for time since onset of MS and somatic symptoms as well as time since onset and non-somatic symptoms. Non-somatic symptoms were more linked to anxiety early in the disease and somatic symptoms were more prominently linked to anxiety later in the disease. Findings suggest that non somatic symptoms of depression and employment predict anxiety in MS	The relationship between different aspects of depression and anxiety may change over the course of the disease
Marrie, Fisk et al. 2015 [6]^a^	Depression, anxiety, bipolar disorder	*n* = 44452 Age: 43.8 ± 13.7 Female (*n*): 31757 Canada	ICD-9/-10	The annual incidence of depression per 100,000 persons with MS was 979 while the incidence of anxiety was 638 of bipolar disorder was 328, and of schizophrenia was 60. The incidence and prevalence estimates of all conditions were higher in the MS population than in the matched population. Although the incidence of depression was higher among women than men in both populations, the disparity in the incidence rates between the sexes was lower in the MS population than in the matched population. Incidence rates were stable over time while prevalence increased slightly	Psychiatric comorbidity is common in MS, and more frequently affected the MS population than a matched population

The selected studies are sorted chronologically by year and alphabetically by author name. Types of articles: ^a^Empirical article; ^b^Review article; Definition papers were not included in the table. *n* = sample size; age: mean ± standard deviation; NA = Not applicable/reported

Abbreviations: *BAI* Beck Anxiety Inventory, *BDI–FS* Beck Depression Inventory–Fast Screen, *BDI (-I/-II)* Beck Depression Inventory, *DSM (-III/-IV)* Diagnostic and Statistical Manual of Mental Disorders, *FSS* Fatigue Severity Scale, *GAD-7* 7-item Generalized Anxiety Disorder Scale, *HADS (-A/-D)* Hospital Anxiety and Depression Scale (-Anxiety Scale/-Depression Scale), *ICD-9/-10* International Classification of Disease, *IDS-SR*_*30*_ Self-rated Inventory of Depressive Symptomatology, *MINI* Mini–International Neuropsychiatric Interview, *MS* Multiple Sclerosis, *NRS* The Numeric Rating Scale, *PHQ-2/-9* Patient Health Questionnaire, *QoL* quality of life, *SCAN* Structured Clinical Interview, *STAI* State-Trait Anxiety Inventory

**Table 8:** Included references

Author, year	Symptom or disorder category	MS population	Diagnostic assessment tools	Main findings	Comments
Marrie, Reingold et al. 2015 [5]^b^	Depression, anxiety, bipolar disorder	NA	NA	Among population-based studies, the prevalence of anxiety was 21.9%, while it was 14.8% for alcohol abuse, 5.83% for bipolar disorder, 23.7% for depression, 2.5% for substance abuse, and 4.3% for psychosis. The results confirmed that psychiatric comorbidity, particularly depression and anxiety, is common in MS. However, the incidence of psychiatric comorbidity remains understudied	None
Ozdemir et al. 2015 [25]^a^	Depression	*n* = 75 Age-RRMS: 32.7 ± 9.6 Age-SPMS: 31.6 ± 8 Female (*n*): 54 Turkey	POMS, MEQ	Both relapsing – remitting- and secondary progressive-type MS patients scored higher on the depression – dejection and fatigue – inertia scales of the POMS than did healthy individuals. Circadian preferences did not differ significantly between the groups. Only gender was found to be predictive for profile of mood	None
Patten et al. 2015 [68]^a^	Depression	*n* = 152 Age (range): 50 (24–82) Female (%): 77.6 Canada	PHQ-9, PHQ-2, CES-D, HADS-D, SCID	All of the scales performed well, each having an area under the ROC > 90%. For example, the PHQ-9 had 95% sensitivity and 88.3% specificity when scored with a cut-point of 11. This cut-point achieved a 56% positive predictive value for major depression. While all of the scales performed well in terms of their sensitivity and specificity, the availability of the PHQ-9 in the public domain and its brevity may enhance the feasibility of its use	None
Strober et al. 2015 [69]^a^	Depression	Depressed: *n* = 11 Age: 43.8 ± 6.2 Female (*n*): 9 Non-depressed: *n* = 70 Age: 47.6 ± 9.5 Female (*n*): 58 USA	BDI-II, BDI–FS, CMDI, MS-BDI	Results suggest that cutoffs of 4 on the BDI–FS and 23 on the CMDI Mood subscale are most useful when screening for depression in MS, with a sensitivity for both of 100%, while a cutoff of 19 on the BDI–II, a cutoff of 22 on the CMDI Evaluative scale, and a cutoff of 8 on the MS-BDI had high specificities, suggesting they can be used as to assist in diagnosing depression in MS	Comparison of depressed and non-depressed MS patients
Butler et al. 2016 [73]^b^	Anxiety, QoL	NA	HADS, BAI, PHQ, Hopkins Symptom Checklist, HAM-A, STAI, GHQ-28/-30, Yale-Brown Obsessive Compulsive Scale, NPI, Health Anxiety Inventory, POMS, MHI, SPIN, DASS, CCEI, NAS, SAS, ZARS, SCL-90, PROMIS, DSM, ICD, K-SADS	Anxiety was strongly associated with both high level of disability and low QoL. A strong association between anxiety and depression was also found. High levels of anxiety exist among persons with MS and highlighting the need for early intervention and treatment of anxiety throughout the course of MS. Anxiety in MS is associated with a number of physical, cognitive, social and psychological factors which have been conceptualized in a model of anxiety. Due to the high levels of comorbidity with depression, many persons with MS are likely to benefit from psychological and pharmacological interventions targeting both anxiety and depression	Given the overlap between anxiety and depression, a transdiagnostic treatment approach is suggested

The selected studies are sorted chronologically by year and alphabetically by author name. Types of articles: ^a^Empirical article; ^b^Review article; Definition papers were not included in the table. *n* = sample size; age: mean ± standard deviation or mean (range); NA = Not applicable/reported

Abbreviations: *BAI* Beck Anxiety Inventory, *BDI–FS* Beck Depression Inventory–Fast Screen, *BDI (-I/-II)* Beck Depression Inventory, *CCEI* Crown-Crisp Experiential Index, *CES-D (-10)* Center for Epidemiologic Studies Depression Scale (10-item scale, identical to the CES-D), *DASS* Depression Anxiety Stress Scale, *DERS* Difficulties in Emotion Regulation Scale, *DSM(-III/-IV)* Diagnostic and Statistical Manual of Mental Disorders, *GAD-7* 7-item Generalized Anxiety Disorder Scale, *GHQ (-28/-30)* General Health Questionnaire, *HADS (-A/-D)* Hospital Anxiety and Depression Scale (-Anxiety Scale/-Depression Scale), *HAM-A* Hamilton Rating Scale for Anxiety, *ICD-9/-10* International Classification of Disease, *K-SADS* Kiddie-Schedule for Affective Disorders and Schizophrenia Present and Lifetime, *MEQ* Morningness-Eveningness Questionnaire, *MHI* Mental Health Inventory, *MS* Multiple Sclerosis, *MS-BDI* MS Specific BDI, *NAS* Nottingham Adjustment Scale, *NPI* Neuropsychiatric Inventory, *PHQ-2/-9* Patient Health Questionnaire, *POMS* Profile of Mood States, *PROMIS-D-8* Patient Reported Outcome Measurement Information System Depression 8-item bank, *QoL* Quality of life, *RRMS* Relapsing–remitting MS, *SAS* Self-Rating Anxiety Scale, *SCID (-IV)* Structured Clinical Interview for the (for DSM-IV), *SCL-90* Symptom-Checkliste, *SPIN* Social Phobia Inventory, *SPMS* Secondary progressive MS, *STAI* State-Trait Anxiety Inventory, *ZARS* Zung Self-Rating Anxiety Scale

**Table 9:** Included references

Author, year	Symptom or disorder category	MS population	Diagnostic assessment tools	Main findings	Comments
Duncan et al. 2016 [48]^a^	Euphoria	*n* = 100 Age: 44.5 ± 11.2 Female (*n*): 86 South Africa	using the classical and contemporary measure and definition of euphoria	The MS group demonstrated high frequencies of euphoria using the classical measure but low frequencies using the contemporary measure and definition. The matched control group demonstrated significantly higher rates than the MS group using the classical measure and lower rates than the MS group using the contemporary measure. The discrepancies in incidence rates of euphoria noted in the literature do not reflect a change in the incidence of euphoria in MS, but rather in the definition and operationalization of “euphoria”	Furthermore, these results highlight the importance of characterizing what represents pathological euphoria as well as the need for better definitions and instruments of measure
Hanna et al. 2016 [51]^a^	Pseudobulbar affect	*n* = 153 Age: 45.6 ± 8.1 Female (*n*): 119 Canada	MACFIMS, CNS-LS, HADS	MS subjects positive for pathological laughing and crying on the CNS-LS but without depression had lower scores on the controlled oral word association test, a measure of verbal fluency, and the California verbal learning test—2 immediate recall score, a verbal memory measure. This study demonstrates a connection between cognitive impairment, specifically verbal fluency and verbal learning, and pathological laughing and crying in MS subjects	Further studies are warranted to explore the causative relationship between cognitive impairment and pathological laughing and crying
Hasselmann et al. 2016 [31]^a^	Depression	*n* = 139 Age: 43.7 ± 10.7 Female (*n*): 101 Germany	BDI-II, ICD-10	Comparisons on a whole-group level produced the expected differences along somatic/non- somatic symptoms. However, when appropriately controlling for depression severity, age, and sex, only four items contributed differentially to BDI-II total scores in MS versus MDD. Depression construct is essentially identical in both groups. The clinical phenotype of “idiopathic” MDD and MS-associated depression appears similar when adequately examined	The relevance of psychotherapeutic approaches for MS- associated depression should be explored in future studies
Hind et al. 2016 [66]^b^	Depression	NA	CES-D (-10), BDI-I/-II, BDI-FS, CMDI, DSM, HADS, mBDI, MeSH, PHQ-9, PROMIS-D-8, YSQ	Twenty-one studies (N = 5,991 Patients with MS) evaluating 12 instruments were included in the review. Risk of bias varied greatly between instrument and validity domain. Well-conducted evaluations of some instruments are unavailable for some validity domains. This systematic review provides an evidence base for trade-offs in the selection of an instrument for assessing self-reported symptoms of depression in research or clinical practice involving people with MS	Detailed and specific recommendations for where further research is needed
Hoang et al. 2016 [38]^a^	Anxiety	*n* = 5084 Age: NA Female (*n*): NA Denmark	ICD-10	MS patients have increased risk of depression and anxiety in both the pre- and the post-diagnostic period and the use of TCAs and SSRIs is higher than in the control population	None
Litster et al. 2016 [76]^b^	Anxiety	NA	HADS-A, BAI, GAD-7, DSM-IV, SCAN	The criterion validity of three screening tools was assessed: the Hospital Anxiety and Depression Scale–Anxiety (HADS-A), Beck Anxiety Inventory (BAI), and 7-item Generalized Anxiety Disorder Scale (GAD-7). The HADS-A was validated against the Structured Clinical Interview for DSM-IV, the Sched- ules for Clinical Assessment in Neuropsychiatry (SCAN) interview, and the BAI. The BAI was validated against the SCAN, and the GAD-7 was validated against the HADS-A. The HADS-A had higher measures of sensitivity and specificity than did the BAI and the GAD-7	Based on this small sample, the HADS-A shows promise as an applicable measure for people with MS. Screening scales used to identify anxiety in MS must be validated against appropriate reference standards

The selected studies are sorted chronologically by year and alphabetically by author name. Types of articles: ^a^Empirical article; ^b^Review article; Definition papers were not included in the table. *n* = sample size; age: mean ± standard deviation; NA = Not applicable/reported

Abbreviations: *BAI* Beck Anxiety Inventory, *BDI–FS* Beck Depression Inventory–Fast Screen, *BDI (-I/-II)* Beck Depression Inventory, *CES-D (-10)* Center for Epidemiologic Studies Depression Scale (10-item scale, identical to the CES-D), *CMDI* Chicago Multiscale Depression Inventory, *CNS-LS* Center for Neurologic Study Emotional Lability Scale, *DSM(-III/-IV)* Diagnostic and Statistical Manual of Mental Disorders, *GAD-7* 7-item Generalized Anxiety Disorder Scale, *HADS (-A/-D)* Hospital Anxiety and Depression Scale (-Anxiety Scale/-Depression Scale), *ICD-9/-10* International Classification of Disease, *mBDI* Modified Beck Depression Inventory, *MACFIMS* Minimal Assessment of Cognitive Function in MS, *MDD* Major Depressive Disorder, *MeSH* Medical Subject Headings, *MS* Multiple Sclerosis, *PHQ-2/-9* Patient Health Questionnaire, *PROMIS-D-8* Patient Reported Outcome Measurement Information System Depression 8-item bank, *QoL* quality of life, *SCAN* Structured Clinical Interview, *YSQ* Yale Single Question

**Table 10:** Included references

Author, year	Symptom or disorder category	MS population	Diagnostic assessment tools	Main findings	Comments
Roy et al. 2016 [13]^a^	Depression, euphoria	*n* = 117 Age: 46.4 ± 9.2 Female (*n*): 61 USA	NEO-FFI, BDI-FS, FSS, MSNQ, NPI, SIP	Personality changes and neuropsychiatric symptoms are found in multiple sclerosis (MS), but no study has evaluated decline compared to healthy controls. This study assessed personality traits and neuropsychiatric symptoms over 3 years using the NEO Five Factor Inventory and the Neuropsychiatric Inventory. Additional metrics evaluated ambulation, manual dexterity and cognitive function. Contrary to hypothesis, patients showed no significant change in personality or neuropsychiatric status relative to controls. Patients were impaired in motor and cognitive function at baseline and follow-up, but showed only slowing in ambulation over time	The findings indicate that neuropsychiatric status is stable in MS over 3 years
Théaudin et al. 2016 [30]^a^	Depression, anxiety	*n* = 711 Age: 44.8 ± 10.3 Female (*n*): 489 Canada	HADS	Notable gender differences included a higher frequency of primary progressive MS in males, higher HADS anxiety scores in females, but no differences in HADS depression scores. In MS, gender influences the frequency of anxiety only. Etiological factors underpinning anxiety and depression in MS are not only different from one another, but also in the case of depression, different from those observed in general population samples	None
Boeschoten et al. 2017 [35]^b^	Depression, anxiety	NA	HADS, BDI, CES-D, PHQ-9	Fifty-eight articles with a total sample size of 87,756 MS patients were selected. Pooled mean prevalence was 30.5% for depression, and 22.1 for anxiety. Prevalence of clinically significant depressive or anxiety symptoms was higher (35% and 34%) compared with disorders (21% and 10%). Prevalence of a depressive disorder was relatively lower in studies from Europe. Anxiety disorder was more prevalent in community-based samples. Sources of high heterogeneity were not revealed. Data of a large number of patients indicate increased prevalence of depression and anxiety in MS	Further research is needed to identify sources of heterogeneity. Issues to consider are the definition of depression and anxiety, patient recruitment, and patient characteristics
Brokate et al. 2017 [24]^a^	Depression	*n* = 231 Age: 42.1 ± 12.6 Female (*n*): NA Germany	BDI, TAP-Alertness	The identification of specific symptoms and grouping depressive symptoms seems to be of special interest. In addition, variable reaction time parameters are included because a slowing down of several patient groups can be expected. We compare 231 patients and 31 control patients concerning the Beck Depression Inventory and the Alertness Task. The structure of depressive symptoms of the groups varies significantly, in that the intensity of symptoms in depressive patients is markedly higher. Furthermore, depressive patients show a considerably lower information processing speed compared to other groups	This retrospective study illuminates the question, whether depressed patients, alcohol dependent patients, schizophrenic patients, and MS patients show a similar structure of depressive symptoms
Flachenecker et al. 2017 [3]^a^	QoL	*n* = 5475 Age: 51.8 ± 11.0 Female (%):74.3 Germany	HRQoL	In all, 84% were below retirement age, and of these, 51% were employed. Employment was related to disease severity, and MS affected productivity at work for 80% of patients. Overall, 96% and 78% of patients experienced fatigue and cognitive difficulties as a problem, respectively. The mean utility and total annual costs were 0.786 and 28,200 € at Expanded Disability Status Scale (EDSS) 0–3, 0.586 and € 44,000 at EDSS 4–6.5 and 0.273 and € 62,700 at EDSS 7–9, respectively. The mean cost of a relapse was estimated at € 2500	Results provide current health economic data on MS in Germany that are important for the development of health policies and for estimating the value of the current and future treatments
Marrie, Walld et al. 2017 [9]^a^	Depression, anxiety	*n* = 3514 Age: 40.8 ± 12.5 Female (*n*): 2544 Canada	ICD-9/-10	Using population‑based administrative (health claims) data from Manitoba, Canada identified 1922 persons with incident MS from 1989 to 2012, and 11,392 age, sex and geographically‑matched controls from the general population. As compared to controls, MS patients had an elevated annual prevalence ratio of depression, and anxiety disorders. The annual prevalence of depression in our matched cohort was similar to that observed in the 2012 Canadian Community Health Survey, although the annual prevalence of anxiety was slightly higher	Administrative data can be used to estimate the annual period prevalence of psychiatric disorders in MS

The selected studies are sorted chronologically by year and alphabetically by author name. Types of articles: ^a^Empirical article; ^b^Review article; Definition papers were not included in the table. *n* = sample size; age: mean ± standard deviation; NA = Not applicable/reported

Abbreviations: *BDI (-I/-II)* Beck Depression Inventory, *BDI-FS* Beck Depression Inventory – Fast Screen, *CES-D (-10)* Center for Epidemiologic Studies Depression Scale (10-item scale, identical to the CES-D), *FSS* Fatigue Severity Scale, *HADS (-A/-D)* Hospital Anxiety and Depression Scale (-Anxiety Scale/-Depression Scale), *HRQoL* Health-Related QoL, *HUI* Health Utilities Index, *ICD-9/-10* International Classification of Disease, *MS* Multiple Sclerosis, *MSNQ* Multiple Sclerosis Neuropsychological Questionnaire, *NEO-FFI* NEO Five Factor Inventory, *NPI* Neuropsychiatric Inventory, *PHQ-2/-9* Patient Health Questionnaire, *QoL* quality of life, *SIT* Sickness Impact Profile, *TAP* Testbatterie zur Aufmerksamkeitsprüfung

**Table 11:** Included references

Author, year	Symptom or disorder category	MS population	Diagnostic assessment tools	Main findings	Comments
Murphy et al. 2017 [12]^b^	Depression, anxiety, bipolar disorder, pseudobulbar affect, euphoria	NA	BDI, CIL, CIS, HADS, HDRS, MADRS, MMSE, NPI, PSE, SAS, SDS, ZSRDS, SSSI	Neuropsychiatric signs and symptoms occur frequently in individuals with MS (MS), either as the initial presenting complaint prior to a definitive neurological diagnosis or more commonly with disease progression. However, the pathogenesis of these comorbid conditions remains unclear, and it remains difficult to accurately elucidate if neuropsychiatric symptoms or conditions are indicators of MS illness severity. Furthermore, both the disease process and the treatments of MS can adversely impact an individual’s mental health	A discussion of the common neuropsychiatric syndromes that occur in MS and describe the clinical symptoms, aetiology, neuroimaging findings and management strategies for these conditions
Patten et al. 2017 [28]^b^	Depression	NA	NA	Depressive disorders occur in up to 50% of people living with MS (MS). Prevalence estimates are generally 2–3-times higher than those of the general population. Myriad aetiologic factors may contribute to the aetiology of depression in MS including biological mechanisms, as well as the stressors, threats, and losses that accompany living with an unpredictable and often disabling disease. Some prominent risk factors for depression such as (younger) age, (female) sex, and family history of depression are less consistently associated with depression in MS than they are in the general population	Management of depression in MS has not been well studied, but available data on detection and treatment align with general principles of depression management While the validity of standard measurement scales has often been questioned, available evidence suggests that standard scales provide valid ratings
Disanto et al. 2018 [14]^a^	Depression, anxiety	*n* = 49652 Age (range): 47 (39–57) Female (*n*): 7308 United Kingdom	NA	MS patients had significantly higher risk of presenting up to 10 years prior to index date with gastric, intestinal, urinary, and anorectal disturbances, anxiety, depression, insomnia, fatigue, headache, and various types of pain. MS risk progressively increased with each additional symptom presented (0–2 years; 2–5 years; 5–10 years). Sensitivity analyses in patients with age at index < 40 years and no neurological disturbances prior to symptoms of interest showed consistent results. Various clinical disturbances precede MS diagnosis by several years, supporting a prodromal phase to the disease and improving our clinical knowledge of early MS	Integrating these symptoms in the diagnostic procedure may help earlier disease identification
Marrie, Patten et al. 2018 [20]^a^	Depression, anxiety, QoL	*n* = 863 Age: 48.6 ± 11.3 Female (*n*): 648 Canada	HRQOL	Lifetime prevalence estimates for depression were approximately 30% regardless of methods used, but 35.8% with current depressive symptoms were not captured by either administrative data or self- reported diagnoses. Prevalence estimates of anxiety ranged from 11 to 19%, but 65.6% with current anxiety were not captured by either administrative data or self-reported diagnoses. Previous diagnoses did not decrease HRQOL after accounting for current symptoms	Depression and, to a greater extent, anxiety remain underdiagnosed and undertreated in MS; both substantially contribute to reduced HRQOL in MS
Marrie, Zhang et al. 2018 [65]^a^	Depression, anxiety	*n* = 253 Age: 51.0 ± 12.9 Female (*n*): 206 Canada	SCID, PHQ-2/-9, HADS, PROMIS, Kessler-6, GAD-7, OASIS	The SCID classified 10.3% with major depression and 14.6% with generalized anxiety disorder. Among the depression measures, the PHQ-9 had the highest sensitivity (84%). Specificity was generally higher than sensitivity and was highest for the HADS-D with a cut-point of 11 (95%). In ROC analyses the area under the curve did not differ between depression measures. Among the anxiety measures, sensitivity was highest for the HADS-A with a cut-point of 8 (82%). Specificity ranged from 83 to 86% for all measures except the HADS-A with a cut-point of 8 (68%). The AUC did not differ between anxiety measures	Overall, performance of the depression and anxiety screening measures was very similar, with reasonable psychometric properties for the MS population, suggesting that other factors such as accessibility and ease of use could guide the choice of measure in clinical practice

The selected studies are sorted chronologically by year and alphabetically by author name. Types of articles: ^a^Empirical article; ^b^Review article; Definition papers were not included in the table. *n* = sample size; age: mean ± standard deviation; NA = Not applicable/reported

Abbreviations: *BDI (-I/-II)* Beck Depression Inventory, *CIS* Clinical Interview Schedule, *HADS (-A/-D)* Hospital Anxiety and Depression Scale (-Anxiety Scale/-Depression Scale), *HDRS* Hamilton Depression Rating Scale, *HRQoL* Health-Related QoL, *MADRS* Montgomery and Asberg Depression Scale, *MMSE* Mini-Mental State Examination, *MS* Multiple Sclerosis, *NPI* Neuropsychiatric Inventory, *OASIS* Overall Anxiety Severity and Impairment Scale, *PROMIS-D-8* Patient Reported Outcome Measurement Information System Depression 8-item bank, *PSE* Present State Examination, *QoL* quality of life, *SAS* Self-Rating Anxiety Scale, *SCID (-IV)* Structured Clinical Interview for the (for DSM-IV), *SDS* Self-rated Depression Scale, *SSSI* Social Stress and Support Interview, *STAI* State-Trait Anxiety Inventory, *ZSRDS* Zung Self-Report Depression Scale

**Table 12:** Included references

Author, Year	Symptom or disorder category	MS population	Diagnostic assessment tools	Main findings	Comments
McKay et al. 2018 [60]^a^	QoL	*n* = 2312 Age at MS symptom onset: 36.9 ± 13.6 Female (*n*): 1751 Canada	GEE, ICD-9/-10	A total of 2312 incident cases of adult-onset MS were followed for a mean of 10.5 years, during which time 35.8% met criteria for a mood or anxiety disorder. The presence of a mood or anxiety disorder was associated with a higher EDSS score (adjusted for disease duration and course, age, sex, socioeconomic status, physical comorbidity count, and disease-modifying therapy exposure). Findings were statistically significant among women, but not men. Presence of psychiatric comorbidities, which were common in our incident MS cohort, increased the severity of subsequent neurologic disability	Optimizing management of psychiatric comorbidities should be explored
Roy et al. 2018 [19]^a^	NA	*n* = 82 Age: 45.9 ± 8.1 Female (*n*): 65	NEO-FFI, BICAMS	Patients were classified as “Cog Stable” or “Cog Decline” based on cognitive deterioration over 5 years. Extraversion and Conscientiousness declined across pooled groups. Follow-up of a group by time interaction found that decline in these traits was more evident in the Cog Decline group, demonstrating a link between personality and cognitive change	In addition to Extraversion and Conscientiousness cognitive status was assessed
Binzer et al. 2019 [59]^a^	Depression, QoL	*n* = 5875 Age at MS symptom onset: 35.1 ± 11.4 Female (*n*): 1751 Sweden	ICD-9/10	Persons with depression were at a significantly higher risk of reaching sustained EDSS scores of 3.0, 4.0, and 6.0, respectively. A similar increased risk among persons exposed to antidepressants was observed, for sustained EDSS scores of 3.0, 4.0, and 6.0, respectively. Persons with MS and comorbid depression had a significantly increased risk of disability worsening	Findings highlights the need for early recognition and appropriate treatment of depression in persons with MS
Patrick et al. 2019 [71]^b^	Depression QoL	NA	PHQ-9	Seven relevant studies were identified, these were of high quality and included 5080 participants from all MS disease-course groups. Strong evidence was found supporting the validity of the PHQ-9 as a unidimensional measure of depression. Used as a screening tool for major depressive disorder (MDD) with a cut-point of 11, sensitivity was 95% sensitivity and specificity 88.3%. Alternative scoring systems that may address the issue of overlap between somatic features of depression and features of MS perse are being developed, although their utility remains unclear. However, data on reliability was limited, and no specific evidence was available on test–retest reliability, responsiveness, acceptability, or feasibility	The PHQ-9 represents a suitable tool to screen for Major Depression. However, use as a diagnostic tool cannot currently be recommended, and the potential value for monitoring depressive symptoms cannot be established without further evidence on test–retest reliability, responsiveness, acceptability, and feasibility
Prakash et al. 2019 [55]^a^	Depression, anxiety, QoL	*n* = 100 Age: 45.5 ± 9.49 Gender (%):85.0 USA	HADS, DERS, WLS, WHOQOL-BREF, CERA	Persons with MS had higher scores on depression, endorsed greater difficulty regulating emotions, and reported lower health related QoL compared with community controls. Higher scores on both measures of depression and anxiety were associated with difficulties in emotion regulation and greater use of maladaptive emotion regulation strategies. Additionally, emotion dysregulation—quantified via use of maladaptive strategies and difficulties in regulating emotions—mediated the effect of MS on symptoms of depression	Emotion dysregulation is associated with symptoms of depression and anxiety

The selected studies are sorted chronologically by year and alphabetically by author name. Types of articles: ^a^Empirical article; ^b^ Review article; Definition papers were not included in the table. *n* = sample size; age: mean ± standard deviation; NA = Not applicable/reported

Abbreviations: *BICAMS* Brief International Cognitive Assessment for MS, *CERA* Contextual Emotion Regulation Assessment, *DERS* Difficulties in Emotion Regulation Scale, *GEE* Generalized Estimating Equations, *HADS (-A/-D)* Hospital Anxiety and Depression Scale (-Anxiety Scale/-Depression Scale), *ICD-9/-10* International Classification of Disease, *MS* Multiple Sclerosis, *NEO-FFI* NEO Five-Factor Inventory, *PHQ-2/-9* Patient Health Questionnaire, *QoL* quality of life, *WHOQOL-BREF* World Health Organization QoL-BREF, *WLS* Satisfaction with Life Scale

**Table 13:** Included references

Author, year	Symptom or disorder category	MS population	Diagnostic assessment tools	Main findings	Comments
Wijnands et al. 2019 [15]^a^	Depression	*n* = 13951 Age: 43.0 ± 6.7 Gender (*n*): 10156 Canada	ICD-10	The administrative and clinical cohorts included 13,951/66,940 and 3202/16,006 people with and without MS (cases/controls). Compared to controls, in the 5 years before the first demyelinating claim or symptom onset, cases had more physician and hospital encounters (per ICD-10 chapter and per physician specialty) for the nervous, sensory, musculoskeletal, and genitourinary systems. Cases had more psychiatrist and urologist encounters, and higher proportions of musculoskeletal, genitourinary or hormonal-related prescriptions (1.1–1.5 times higher). However, cases had fewer pregnancy-related encounters than controls	Phenotyping the prodrome 5 years before clinical recognition of MS is feasible
Sparaco et al. 2019 [79]^b^	Depression, anxiety, bipolar disorder, QoL	NA	BDI-II, CMDI, HADS, CES-D, PHQ-9, PROMIS, HDRS,	Epidemiological and clinical aspects of psychiatric syndromes in MS as well as self-report diagnostic scales and radiological correlates of psychiatric syndromes in MS are described. Moreover, some radiological studies about primary psychiatric disorders are reported to underline how gray matter atrophy, white matter abnormalities and corpus callosum involvement in these diseases, as features in common with MS, may explain the more frequent occurrence of psychiatric syndromes in MS than in the general population	None
Erlangsen et al. 2020 [53]^a^	QoL	*n* = 31136 Age: NA Female (*n*): NA Denmark	ICD-10	There was a significantly higher rate of suicide among those with a diagnosed neurological disorder (includes MS) than all other persons. People diagnosed with MS had an adjusted IRR of 1.7 (95% CI, 1.6–1.7). Diagnosis of a neurological disorder was associated with a small but statistically significant increased risk of death by suicide	The retrospective cohort study included persons in Denmark from 1980 through 2016
Fidao et al. 2021 [54]^a^	Depression, QoL	*n* = 2104 Age: 45.6 ± 10.5 Female (*n*): 1728 Australia	MSQOL-54, MHC-SF	The median mental QoL score was 71.9/100. The mean fatigue score was 41.5/63, with 65.6% participants having clinically significant fatigue. In the SEM evaluating depression as a mediator of the fatigue-QoL relationship, mental QoL was 14.72 points lower in participants with clinically significant fatigue, of which depression accounted for 53.0%. In the SEM evaluating physical activity as a mediator of the fatigue-QoL relationship, mental QoL was 10.89 points lower in participants with clinically significant fatigue, of which the indirect effect via physical activity accounted for only 4.4%. Depression accounted for the majority of the fatigue-mental QoL relationship when modelled as a mediator, while physical activity had only a minor role	Findings may inform the development of treatments for reducing the impacts of fatigue and improving mental QoL in patients with MS
Filser et al. 2021 [80]^a^	Depression, anxiety	*n* = 314 Age: 48.4 ± 10.8 Female (*n*): 232 Germany	MeSyMS	MeSyMS revealed an excellent internal consistency. Compared to control subjects, MS patients showed significant mental health problems in all three dimensions (depression, anxiety, and social and emotional health problems). In comparison to the subscales (depression and anxiety), the dimension of social and emotional health problems revealed the highest accuracy and turned out to be the only scale that reliably differentiated between the groups	Social and emotional health problems turned out to be the most important aspect when identifying disease-related mental health symptoms in MS

The selected studies are sorted chronologically by year and alphabetically by author name. Types of articles: ^a^Empirical article; ^b^Review article; Definition papers were not included in the table. n = sample size; age: mean ± standard deviation; NA = Not applicable/reported

Abbreviations: *BDI (-I/-II)* Beck Depression Inventory, *CES-D (-10)* Center for Epidemiologic Studies Depression Scale (10-item scale, identical to the CES-D), *CMDI* Chicago Multiscale Depression Inventory, *HADS (-A/-D)* Hospital Anxiety and Depression Scale (-Anxiety Scale/-Depression Scale), *HDRS* Hamilton Depression Rating Scale, *ICD-9/-10* International Classification of Disease, *MeSyMS* Mental symptoms in MS, *MHC-SF* Mental Health Continuum Short Form, *MS* Multiple Sclerosis, *MSQOL-54* Multiple Sclerosis Quality of Life-54, *PHQ-2/-9* Patient Health Questionnaire, *PROMIS-D-8* Patient Reported Outcome Measurement Information System Depression 8-item bank, *QoL* quality of life
